# Protective Effects of Defatted Sticky Rice Bran Extracts on the Early Stages of Hepatocarcinogenesis in Rats

**DOI:** 10.3390/molecules24112142

**Published:** 2019-06-06

**Authors:** Aphisit Dokkaew, Charatda Punvittayagul, Orapin Insuan, Pornngarm Limtrakul (Dejkriengkraikul), Rawiwan Wongpoomchai

**Affiliations:** 1 Department of Biochemistry, Faculty of Medicine, Chiang Mai University, Chiang Mai 50200, Thailand; abhisitlumduan@gmail.com (A.D.); pornngarm.d@cmu.ac.th (P.L.(D.)); 2 Research Affairs, Faculty of Veterinary Medicine, Chiang Mai University, Chiang Mai 50100, Thailand; charatda.pun@cmu.ac.th; 3 Department of Medical Technology, School of Allied Health Sciences, University of Phayao, Phayao 56000, Thailand; orapin.th@up.ac.th; 4 Anticarcinogenesis and Apoptosis Research Cluster, Faculty of Medicine, Chiang Mai University, Chiang Mai 50200, Thailand; 5 Functional Food Research Center for Well-being, Chiang Mai University, Chiang Mai 50200, Thailand

**Keywords:** diethynitrosamine, glutathione *S*-transferase placental form, rice bran, cancer chemoprevention, hepatocarcinogenesis

## Abstract

Use of natural products is one strategy to lessen cancer incidence. Rice bran, especially from colored rice, contains high antioxidant activity. Cancer chemopreventive effects of hydrophilic purple rice bran extract (PRBE) and white rice bran extract (WRBE) on carcinogen-induced preneoplastic lesion formation in livers of rats were investigated. A 15-week administration of PRBE and WRBE did not induce hepatic glutathione *S*-transferase placental form (GST-P) positive foci formation as the biomarker of rat hepatocarcinogenesis. PRBE and WRBE at 500 mg/kg body weight significantly decreased number and size of GST-P positive foci in diethylnitrosamine (DEN)-initiated rats. The number of proliferating nuclear antigen positive hepatocytes were also reduced in preneoplastic lesions in both PRBE and WRBE fed DEN-treated rats. Notably, the inhibitory effect on GST-P positive foci formation induced by DEN during the initiation stage was found only in rats treated by PRBE for five weeks. Furthermore, PRBE attenuated the expression of proinflammatory cytokines involving genes including TNF-α, iNOS, and NF-κB. PBRE contained a higher number of anthocyanins and other phenolic compounds and vitamin E. PRBE might protect DEN-induced hepatocarcinogenesis in rats via attenuation of cellular inflammation and cell proliferation. Anthocyanins and other phenolic compounds, as well as vitamin E, might play a role in cancer chemopreventive activity in rice bran extract.

## 1. Introduction

Hepatocellular carcinoma (HCC) is the most common type of liver cancer and presents a significant problem, particularly in developing countries [1]. Viral hepatitis B and C infection and carcinogen exposure through alcohol and aflatoxins are major risk factors of HCC development [2]. Cancer can be prevented either by avoiding carcinogen exposure or routine consumption of natural anticarcinogens. Phytonutrients and phytochemicals in fruits, vegetables, cereals, and edible mushrooms have known capabilities to prevent various chronic diseases such as cancer. Natural products and synthetic agents based on phytochemicals are considered a complementary strategy to improve organism resistance to cancer formation [3]. For example, anthocyanins derived from colored vegetables and fruits could improve intracellular antioxidant systems and detoxifying enzyme activities, resulting in reduction of initiated cell formation [4]. Some phenolic acids detected in numerous grains could inhibit cancer cell metastasis by suppressing enzyme matrix metalloproteinase-2 (MMP-2) via Ras/Akt/NF-κB signaling pathway [5]. Flavonoids such as quercetin and kaempferol have been shown to modulate metabolic activation of carcinogen via inhibition of CYP1A1 and AhR functions [6]. Thus, interest in these candidate compounds that can either prevent or inhibit carcinogenesis has increased.

Rice bran is a by-product from the rice milling process and contains several phytonutrients and phytochemicals including vitamins, γ-oryzanol, and phenolic compounds [7]. Many researchers have found that phytochemicals in rice bran presented numerous beneficial effects such as antioxidant activity [8] and anticancer activities in liver, colon, and breast [7]. Defatted rice bran residues retain some precious polar phytochemicals. Anthocyanins, which occur particularly in colored rice, exhibit numerous chemopreventive effects including suppression of enzyme α-glucosidase activity [9], antimutagenicity [10], and anti-inflammatory activities [11]. Our previous studies demonstrated that methanol extract of purple rice seed presented antimutagenicity against AFB_1_-and MeIQ-induced mutagenesis in Salmonella mutation assay [12]. Suwannakul et al. [13] found that methanol extract of purple rice bran reduced micronucleated hepatocytes in aflatoxin B_1_-initiated rats via modulation of carcinogenic metabolizing enzymes. In addition, polar extracts of white rice bran inhibited growth of breast and colon cancer cells [14]. However, there is no evidence concerning a comparative study between purple and white rice brans. Thus, this research aimed to compare chemopreventive effects between defatted brans of purple and white rice against carcinogen-initiated hepatocarcinogenesis in rats and their possible inhibitory mechanism.

## 2. Results 

### 2.1. Phytochemical Constituents in Defatted Rice Bran Extracts 

One kilogram of defatted purple and white rice bran provided 38 ± 11 g of purple rice bran methanol extract (PRBE) and 52 ± 33 g of white rice bran methanol extract (WRBE), respectively. The PRBE contained a greater number of phenolic compounds, including flavonoids, anthocyanins, and tocols, than the WRBE. Major anthocyanins in PRBE were cyanidin 3-glucoside and peonidin 3-glucoside. Protocatechuic acid and vanillic acid were predominantly found in PRBE, while *p*-coumaric and 4-hydroxybenzoic acid were contained in WRBE. The major tocol isoform was γ-tocotrienol. Interestingly, a higher amount of γ-oryzanol was found in WRBE than PRBE (Table 1). 

### 2.2. Effect of 15-Week Administration of Rice Bran Extracts on Promotion Stage of Hepatocarcinogenesis 

**Figure 1:**
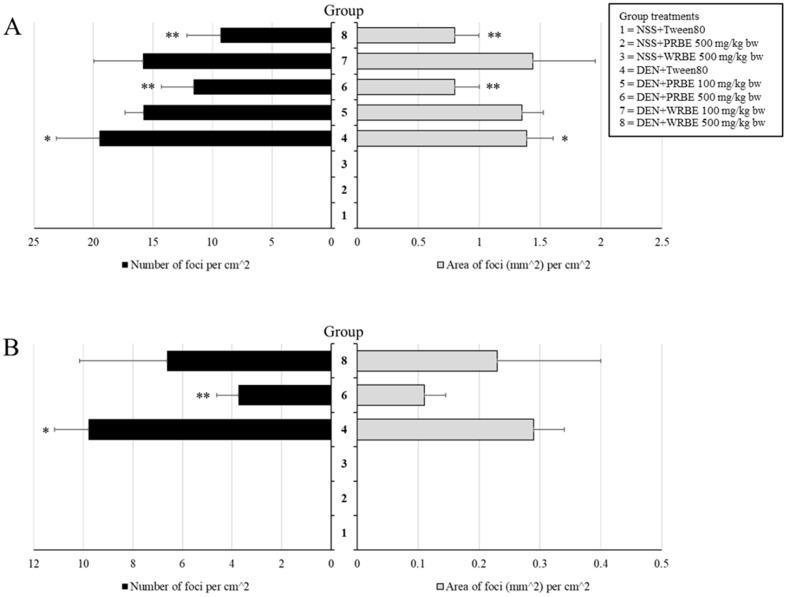
Effect of sticky rice bran extracts on glutathione *S*-transferase placental form (GST-P) positive foci formation of rats, (**A**) 15-week administration rats, (**B**) 5-week administration rats. NSS: Normal saline solution, DEN: Diethylnitrosamine. * Significantly different from group 1 (*p* < 0.05). ** Significantly different from group 4 (*p* < 0.05).

Final body weight of rats treated with diethylnitrosamine (DEN) significantly decreased when compared with the control group. Final body weight also decreased in 500 mg/kg bw PRBE-fed rats; however, food and water intake and relative organ weights did not change in each group (data not shown). Glutathione *S*-transferase placental form (GST-P) positive foci in the livers of both PRBE- and WRBE-treated rats were not detected and extracts did not show carcinogenicity. DEN induced GST-P positive foci formation, while administration of 500 mg/kg bw of PRBE and WRBE suppressed both number and size of hepatic GST-P positive foci in DEN-initiated rats (Figure 1A). 

**Figure 2:**
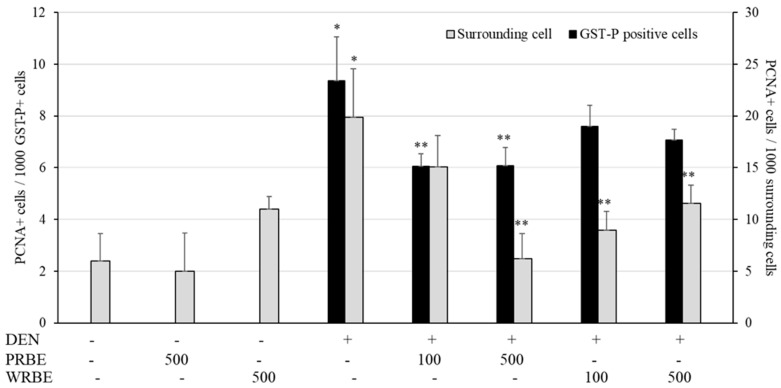
Effect of sticky rice bran extracts on cell proliferation in rat liver of 15-week protocol. NSS: Normal saline solution, DEN: Diethylnitrosamine, PRBE: Purple rice bran extract, WRBE: White rice bran extract. * Significantly different from group 1 (*p* < 0.05). ** Significantly different from group 4 (*p* < 0.05).

Proliferating cell nuclear antigen (PCNA) protein, a cell proliferation biomarker, was markedly detected in the liver of DEN-treated rats. Interestingly, high dose of 500 mg/kg bw of PRBE administration decreased the number of PCNA labeled cells in GST-P positive foci and also in the surrounding areas of the liver of DEN-treated rats. However, WRBE reduced only PCNA positive cells in normal area when compared with positive control. Both extracts did not affect cell proliferation in normal rat livers (Figure 2). 

### 2.3. Effect of Five-Week Administration of Rice Bran Extracts on Initiation Stage of Hepatocarcinogenesis 

Due to the result of the 15-week administration, 500 mg/kg bw concentration of the extracts was chosen for further study on the initiation stage protocol. Although DEN injection decreased the body weight of rats, administration of extracts did not alter final body weight. Food intake, water intake, and relative organ weights did not change (data not shown). PRBE and WRBE treatments did not induce GST-P formation in rats nor indicate hepatocarcinogenicity in rat liver. Interestingly, only treatment of PRBE decreased the number but not the area of hepatic GST-P positive foci in DEN-initiated rats (Figure 1B).

**Figure A1:**
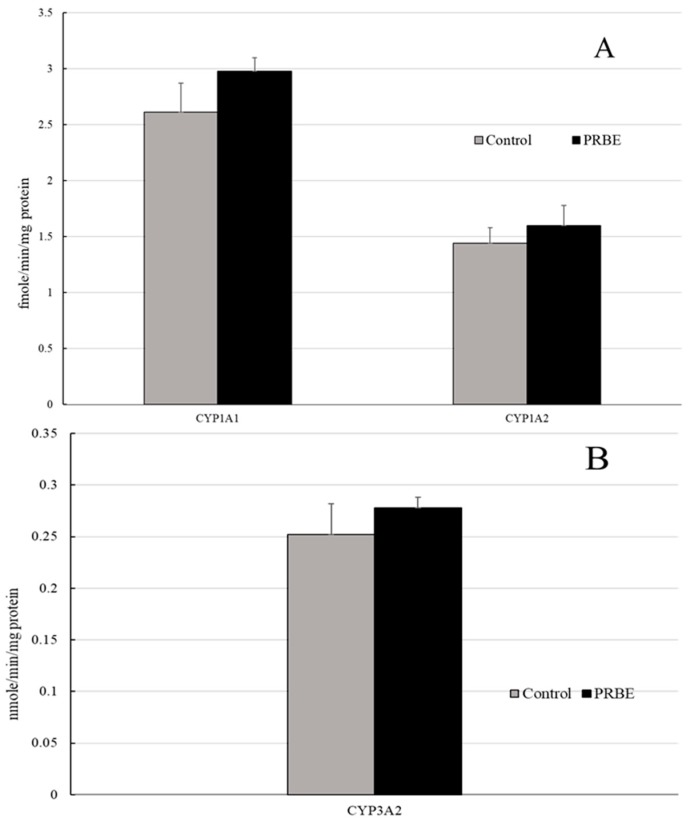
Effect of PRBE on phase I metabolizing enzymes activity in 5-week protocol. (**A**) CYP1A1 and 1A2 activities, (**B**) CYP3A2 activity. The results are shown as mean ± SD. DEN: Diethylnitrosamine, PRBE: Purple rice bran extract, CYP: Cytochrome P450.

**Figure A2:**
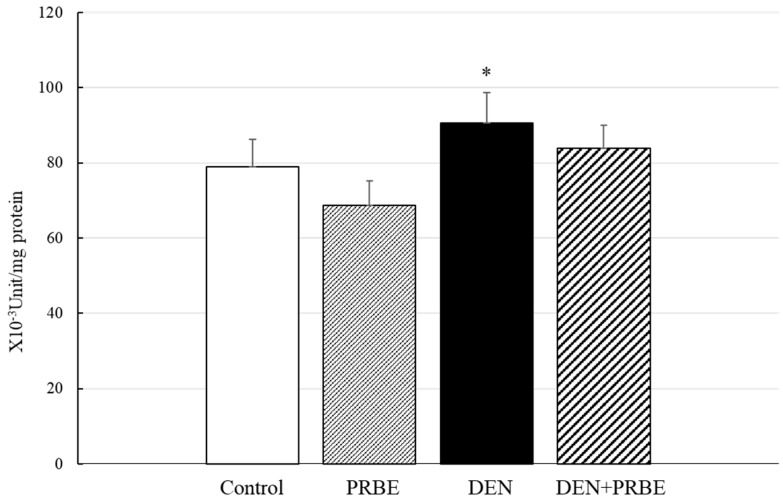
Effect of PRBE on Glutathione *S*-transferase activity in 5-week protocol. The results are shown as mean ± SD. DEN: Diethylnitrosamine, PRBE: Purple rice bran extract. * Significantly different from group 1 (*p* < 0.05).

To investigate the inhibitory mechanism of PRBE on the initiation stage, xenobiotic metabolizing enzymes and inflammation were evaluated. Results showed that activities of cytochrome P450 1A1, 1A2, and 3A2 were not modulated when treated with PRBE (Appendix A; Figure A1). Moreover, activity of glutathione *S*-transferase, one of the detoxifying enzymes, did not change in PRBE treated rats (Appendix A; Figure A2). Gene expression of proinflammatory cytokines and their related genes including tumor necrosis factor (TNF)-α, interleukin (IL)-1β, NF-κB, and nitric oxide synthase (iNOS) were also investigated. The expression of these genes was induced in DEN-treated rat groups; however, gene expressions were not altered in the PRBE-treated group. Notably, PRBE attenuated the expression of TNF-α, NF-κB, and iNOS when compared with the DEN-treated group (Table 2).

## 3. Discussion

Certain phytonutrients and phytochemicals found in plants have been accepted as cancer chemopreventive agents in daily life. They are able to either block or retard entire steps of carcinogenesis. Our findings showed that defatted rice bran extracts reduced preneoplastic GST-P positive focal development in the liver of DEN-initiated rats. A 15-week administration of both purple and white rice bran extracts at high dose during DEN exposure decreased number and size of hepatic GST-P positive foci in rats. However, anticarcinogenicity of white rice bran extract was not observed during the first five weeks of the experiment. Thus, purple rice bran displayed greater cancer chemopreventive potential than white rice bran, possibly caused by lower polyphenol and vitamin E contents in white rice bran. Although γ-oryzanol level was higher in white rice bran than purple rice bran, the amount of γ-oryzanol presented here did not act on the initiation stage of DEN-induced hepatocarcinogenesis in rats. 

Diethylnitrosamine (DEN) is a classical hepatocarcinogen commonly used in rodent models, providing related histopathogenic consequences to human liver cancer. The ultimate form of DEN produced from metabolic activation can cause DNA alkylation and oxidative damage, which disrupt cell proliferation and apoptosis in hepatocytes leading to precancerous and cancerous formation [15]. GST-P positive foci in the liver have been accepted as preneoplastic lesions in rat liver [16]. We found that sticky rice bran extracts reduced GST-P positive foci formation in the liver of DEN-initiated rats. It is well known that one cancer preventive mechanism involves the alteration of cancer cell numbers by either reducing cell proliferation or inducing apoptosis [3]. Purple rice bran extract reduced numbers of PCNA, a marker of cell proliferation, in both preneoplastic lesions and the surrounding areas during the promotion stage of DEN induced hepatocarcinogenesis. Our results affirmed that purple rice bran extract presented stronger cancer chemopreventive effects than white rice bran extract.

Chronic inflammation involving NF-κB, MAPK, and STAT pathways is considered as a critical factor contributing to cellular transformation and tumor development [17]. We also found that repeated DEN exposure increased NF-κB expression, leading to activation of various pro-inflammatory cytokines, which concurred with other groups [18]. TNF-α plays an important role in hepatocarcinogenesis by modulation of downstream signaling such as NF-κB, p38 MAPK, and Jun-(N)-terminal kinase (JNK) pathways [19]. Limtrakul and group [20] found that extracts of Thai black rice exhibited suppression of inflammatory responses by down-regulation of NF-κB and AP-1 signaling pathways in macrophages. Furthermore, iNOS also plays a crucial role in hepatocellular carcinoma development via interaction with NF-κB and Ha-RAS/extracellular signal-regulated kinase (ERK) [21]. Our study determined that PRBE attenuated the expression of NF-κB, TNF-α, and iNOS in the liver of DEN-induced rats. Consequently, we suggest that protective effects of PRBE in DEN-induced preneoplastic lesion formation in the liver occurred through the attenuation of pro-inflammatory cytokines involved in the NF-κB pathway. 

PRBE, which exhibited stronger cancer chemopreventive potential than WRBE, contained higher content of vitamin E and phenolic compounds especially anthocyanins. Several studies have demonstrated that anthocyanin-rich extracts from colored fruits and vegetables presented chemopreventive effects against various chemically induced stages of hepatocarcinogenesis in rats. Their inhibitory mechanisms could be involved with the modulation of antioxidant systems and the induction of apoptotic pathways, as well as suppression of the inflammatory response through modulation of the NF-κB signaling pathway [22,23,24,25]. Anthocyanins might act as cancer chemopreventive agents in defatted purple rice bran. Recently, the validation of individual anthocyanins on cancer chemopreventive activity in animal models has been limited, which might be due to the high cost and complicated synthesis. Thus, the use of purple rice to deliver anthocyanins might be more pragmatic. Anthocyanins are rarely absorbed in the small intestine, but can be degraded to their aglycones and subsequently converted to protocatechuic acid by colonic microflora, which can then be absorbed into the blood circulation [26,27,28] Protocatechuic acid and other phenolic acid metabolites are also contained in PRBE, which showed anticarcinogenicity in line with previous studies [29,30,31]. Several reports have reviewed the beneficial effects of various vitamin E isoforms on the chemopreventive properties in different types of cancer [32]. Our study found that PRBE contained higher levels of vitamin E, especially tocotrienols, than WRBE. Tocotrienols have been shown to suppress cancer cell proliferation, the induction of cancer cell apoptosis and the modulation of proinflammatory cytokine expression [33,34]. These phytochemicals might be a candidate for anticarcinogens in purple rice bran. In order to confirm the chemopreventive efficacy of PRBE, the chemically induced formation of tumors in the liver and also in different organs in animal models needs to be investigated. Furthermore, no epidemiological reports exist concerning the beneficial effects of purple rice. Thus, further studies are required involving the bioavailability of the active compounds and chemopreventive potential of purple rice bran in humans.

In this study, the cancer chemopreventive dose of PRBE was 500 mg/kg bw, which approximates to 4.865 g for daily intake by humans. The safety of this dose was approved in male rats under administration for 105 days. Furthermore, this dose did not influence the principal xenobiotic metabolizing enzymes including CYP3A2. Activation or inhibition of human CYP3A4 or 3A2 in rats by natural products was frequently found in interaction with certain drugs, resulting in undesired effects from hyperactivity or inactivity [35]. Thus, the ingestion of PRBE, together with particular drugs, such as antihypertensive drugs, may have less adverse effects on phytochemical–drug interaction. These findings contribute to the value of Thai purple rice as a natural alternative for cancer prevention and to further applied research concerning the use of purple rice products as health foods. 

## 4. Materials and Methods

### 4.1. Chemicals 

Diethylnitrosamine (DEN) was obtained from Sigma Aldrich (St. Louis, MO, USA). Anti-rat GST-placental form was obtained from MBL (Nagoya, Japan). Envision^TM^ G/2 Doublestain System, Rabbit/Mouse (DAB+/Permanent Red) was obtained from Dako (Denmark). The phenolic acids, flavonoids, anthocyanins, sodium phytate, γ-oryzanol, and tocol standards for chemicals analysis were high-performance liquid chromatography (HPLC)-grade. All other chemicals were analytical grade.

### 4.2. Preparation of Methanol Extract from Sticky Rice Bran

Rice (*Oryza sativa* L.) was cultivated from August to November 2016 at the Faculty of Agriculture, Chiang Mai University, Thailand. The Kum Doi Saket and Sanpatong varieties used as purple and white sticky rice, respectively, are commonly cultivated in Northern Thailand. After rice milling, the brans were defatted by hexane extraction. The resulting residues were macerated in absolute methanol and the filtrates were evaporated and lyophilized. The purple rice bran extract (PRBE) and white rice bran extract (WRBE) were kept at −20 °C in a refrigerator until required for analysis.

### 4.3. Determination of Chemical Constituents in Sticky Rice Brans Extracts

The Folin–Ciocalteu method and aluminum chloride colorimetric method were used to evaluate total phenolic compounds [36] and flavonoids, respectively [37]. Total phytic acids were evaluated using the colorimetric Wade reagent method [38]. Total anthocyanins were measured using the pH-differential method [39]. The HPLC technique was used to investigate some phenolic acids, flavonoids, and anthocyanins according to Punvittayagul et al. [12]. *p*-Coumaric acid, ferulic acid, gallic acid, protocatechuic acid, vanillic acid, catechin, epicatechin, rutin, quercetin, cyanidin-3-glucoside, cyanidin-3-rutinoside, and peonidin-3-glucoside were used as standards. 

Vitamin E and γ-oryzanol were evaluated by HPLC according to Insuan et al. [40], while γ-oryzanol contents were measured at a UV-visible wavelength of 325 nm under isocratic elution. Vitamin E contents were compared with standard α, β, γ and δ forms of tocopherols and α, β and γ forms of tocotrienols. Fluorescence detection wavelengths were an excitation wavelength of 294 nm and an emission wavelength of 326 nm. 

### 4.4. Anticarcinogenicity of Methanol Extracts of Sticky Rice Bran on DEN-treated Rats

**Figure 3:**
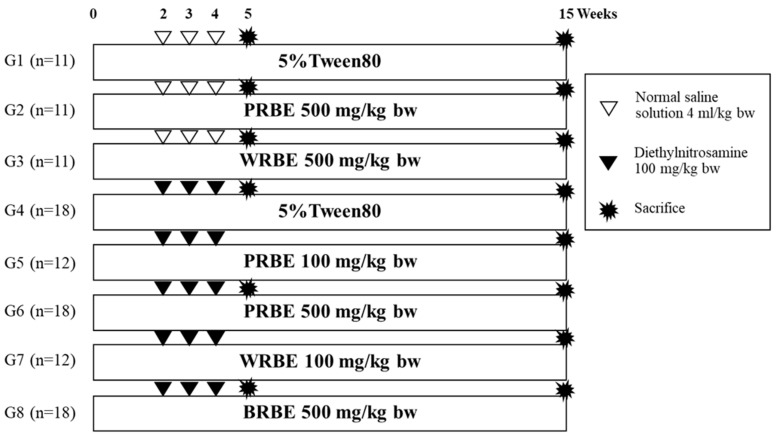
The experimental protocol for studying on diethylnitrosamine induced early stages of hepatocarcinogenesis in rats. PRBE: Purple rice bran extract; WRBE: White rice bran extract.

Three-week-old (90–100 g) male Wistar rats were purchased from the National Laboratory Animal Center, Mahidol University, Nakhon Pathom, Thailand. All rats were housed in stainless steel cages and fed with a pellet diet and tap water ad libitum with temperature control of 25 ± 1 °C under a dark–light cycle. All animal experimental designs were approved by the Animal Ethics Committee of the Faculty of Medicine, Chiang Mai University (20/2560). The rats were separated into 8 groups. Groups 1 to 3 were intraperitoneally injected with normal saline, whereas groups 4 to 8 were intraperitoneally injected with 100 mg/kg bw of DEN at weeks 2, 3, and 4 of the experiments. Groups 1 and 4 served as negative and positive controls, respectively. Two weeks before DEN injection, 100 mg/kg bw of PRBE was orally fed to group 5, while 500 mg/kg bw of PRBE was given to groups 2 and 6. Group 7 was treated with 100 mg/kg bw of WRBE, whereas groups 3 and 8 were fed with 500 mg/kg bw of WRBE. At week 5 of the experiment, every 5th rat of groups 1, 4, 6, and 8 was sacrificed to study the effect of PRBE and WRBE on the initiation stage of hepatocarcinogenesis. The remaining rats were sacrificed at week 15 to evaluate the effect of the extracts on the promotion stage of hepatocarcinogenesis. The treatment procedure is shown in Figure 3. 

### 4.5. Evaluation of Glutathione S-Transferase Placental form Positive Foci in Liver Tissues

The glutathione *S*-transferase placental form (GST-P) positive foci were stained by the avidin–biotin complex method according to Thumvijit et al. [41]. The liver tissues were deparaffinized and rehydrated with xylene and ethanol, respectively, at optimal concentration. To inhibit pseudoperoxidase, 3% hydrogen peroxide was added, while non-specific binding protein was inactivated using 1% skim milk. The specimens were incubated with rabbit polyclonal rat anti-GST-P antibody followed by anti-mouse IgG biotinylated antibody. After that, they were continually incubated with ABC-PO (Rabbit IgG) kit and drenched with diaminobenzidine (DAB). Number and area of GST-P positive foci greater than 0.15 mm^2^ and 0.20 mm^2^ were measured in the 5- and 15-week experiments, respectively, using the LAS Interactive Measurement program.

### 4.6. Determination of Cell Proliferation in Liver Tissues by Double Staining Immunohistochemistry 

To determine the effect of rice bran extract on cell proliferation in preneoplastic lesions in the liver, proliferating cell nuclear antigen (PCNA), a cell proliferation biomarker, was measured in liver tissues obtained from the 15-week experiment. They were performed using the EnVision Doublestain system according to the manufacturer’s recommendations. Firstly, the liver sections were incubated with citrate buffer at 98 °C for 10 min and incubated with H_2_O_2_. After that, the slides were incubated with dual endogenous enzyme block, followed by anti-PCNA antibody and polymer/horseradish peroxidase. The slides were drenched with DAB and the brown color of PCNA protein was observed in the nucleus of the hepatocyte. Then, double stain block was added followed by rat GSTP polyclonal antibody, Rabbit/Mouse link and polymer/alkaline phosphatase (AP). Permanent Red was used as a substrate of AP and the reddish color of GST-P was inspected in the cytoplasm of the hepatocyte. Numbers of PCNA positive hepatocytes labeled in GST-positive foci and the surrounding area were counted under a light microscope.

### 4.7. Determination of Phases I and II Xenobiotic Metabolizing Enzyme Activities

Frozen liver (5-week protocol) was homogenized in homogenizing buffer and continually centrifuged at 10,000× *g* for 20 min at 4 °C. The supernatant was carried to further centrifuge at 100,000× *g* for 1 h at 4 °C. The cytosolic supernatant and the microsomal pellet were measured for total protein content using the Lowry method.

Activities of cytochrome P450 (CYP) 1A1, 1A2, and 3A2 were determined according to Suwannakul et al. [13] using ethoxyresorufin-*O*-deethylation (EROD), methoxyresorufin *O*-demethylation (MROD) and erythromycin *N*-demethylation (ENDM) methods, respectively. Briefly, the reaction mixture containing Tris buffer pH 7.8, NADPH, and EROD for CYP1A1, or MROD for CYP1A2, was added to the microsomal fraction. Activity was measured by a spectrofluorometer at an excitation wavelength of 520 nm and an emission wavelength of 590 nm. For CYP3A2, NADPH was added to the reaction mixture consisting of phosphate buffer saline, erythromycin, and MgCl_2_ and microsomal fraction. After incubation at 37 °C for 20 min, trichloroacetic acid was added to stop the reaction and the mixture was centrifuged. Then, the supernatant was mixed with Nash reagent and incubated at 15 °C for 15 minutes. Activity was measured at a wavelength of 405 nm and results were reported as nmole/min/mg protein. 

Glutathione *S*-transferase (GST) activity was examined using 1-chloro,2,4-dinitrobenzene (CDNB) as a substrate [42]. Liver cytosol was mixed with a reaction mixture that contained glutathione and CDNB and then incubated at 37 °C. The mixture was measured using a spectrophotometer at a wavelength of 340 nm. Results were reported as U/mg protein.

### 4.8. Determination of Pro-Inflammatory Cytokine Gene Expression by Real-time PCR

Total RNA was extracted from rat liver using Purezol^TM^ RNA Isolation Reagent according to the manufacturer’s instruction. cDNA synthesis was performed using high-capacity cDNA reverse transcription kit (Applied Biosystems^®^, Foster City, CA., USA.) following the manufacturer’s manual. Primer lists are shown in Table 3. The qPCR amplification was performed using SensiFAST^TM^SYBR Lo-ROX Kit (Bioline Reagent Ltd, London, UK.) at 95 °C for 52 min, followed by 40 cycles at 95 °C for 5 s, 60 °C for 10 s, and 72 °C for 20 s. Gene expression was normalized with β-actin and quantified using the 2^-^^ΔΔct^ method normalized to the level of β-actin [43].

### 4.9. Statistical Analysis

Results were expressed as mean ± SD. Statistical comparisons between groups were calculated via independent sample *t*-test and one-way analysis of variance (ANOVA) followed by LSD post hoc test. Differences were measured as significant for *p* < 0.05.

## 5. Conclusions

Methanol extract of sticky rice bran was not hepatocarcinogenic in rats. Purple rice bran exhibited stronger cancer chemopreventive action than white rice bran. Anthocyanins and other phenolic compounds, as well as vitamin E, might be the candidate cancer chemopreventive agents in defatted rice bran. The aberration of NF-κB signaling pathway by purple rice bran extract might suppress some pro-inflammatory cytokines like TNF-α and inflammatory enzymes like iNOS, and diminish preneoplastic cell proliferation in DEN-induced early stages of hepatocarcinogenesis in rats.

## Figures and Tables

**Table 1:** The components of phytochemicals in methanol sticky rice bran extracts.

Compounds (Per Gram Extract)	Purple Rice Bran	White Rice Bran
**Spectrophotometry analysis**		
Total phenolic compounds (mg)	74.2 ± 3.6 *	16.5 ± 1.0
Total flavonoids (mg)	50.7 ± 1.4 *	12.7 ± 1.8
Total anthocyanins (μg)	29.7 ± 0.2 *	ND
Total phytic acids (mg)	9.71 ± 2.4	7.39 ± 1.9
**HPLC method**		
Total γ-oryzanol (mg)	3.71 ± 0.02	5.05 ± 0.04 *
Total vitamin E (μg)	321.4 ± 9.3 *	274.7 ± 4.4
α-tocopherol (μg)	24.5 ± 0.3	13.6 ± 0.1
β-tocopherol (μg)	ND	26.1 ± 0.3
γ-tocopherol (μg)	44.1 ± 1.9	39.4 ± 0.3
δ-tocopherol (μg)	5.6 ± 0.1	ND
α-tocotrienol (μg)	14.1 ± 0.1	ND
γ-tocotrienol (μg)	202.6 ± 6.9	167.3 ± 5.1
δ-tocotrienol (μg)	30.5 ± 0.4	28.0 ± 0.2
*p*-Coumaric acid (mg)	0.35 ± 0.00	0.84 ± 0.02 *
Protocatechuic acid (mg)	7.12 ± 0.03 *	ND
Vanillic acid (mg)	4.73 ± 0.02 *	ND
4-Hydroxybenzoic acid (mg)	ND	0.35 ± 0.01 *
Cyanidin-3-glucoside (μg)	5.7 ± 0.0 *	ND
Peonidin-3-glucoside (μg)	3.6 ± 0.0 *	ND

Values are presented as mean ± SD, ND: Not Detected, *n* = 3. * Significantly different between the extract (*p* < 0.05).

**Table 2:** Effect of purple rice bran extract (PRBE) on the expression of pro-inflammatory cytokines related genes in five-week protocol.

Group	Chemical	Treatment	Gene Expression Relative to β-Actin (Fold Change)			
			TNF-α	IL-1β	iNOS	NF-κB
1	NSS	5% Tween80	1.00 ± 0.20	1.00 ± 0.29	1.00 ± 0.40	0.99 ± 0.27
2	NSS	PRBE 500 mg/kg bw	1.00 ± 0.17	0.98 ± 0.15	0.79 ± 0.44	0.99 ± 0.11
3	DEN	5% Tween80	2.31 ± 0.55 *	3.12 ± 1.10 *	7.73 ± 4.56 *	2.10 ± 0.99 *
4	DEN	PRBE 500 mg/kg bw	1.76 ± 0.45 **	2.60 ± 0.78	2.23 ± 1.15 **	0.89 ± 0.13 **

The results are shown as mean ± SD. NSS: Normal saline solution, DEN: Diethylnitrosamine, PRBE: Purple rice bran extract, WRBE: White rice bran extract. * Significantly different from group 1 (*p* < 0.05). ** Significantly different from group 3 (*p* < 0.05).

**Table 3:** The selective primers for evaluation of pro-inflammatory cytokines.

Genes	Forward Primer	Reverse Primer
TNF-α	5′-AAATGGCCCTCTCATCAGTCC-3′	5′-TCTGCTTGGTGGTTTGCTACGAC-3′
IL-1β	5′-CACCTCTCAAGCAGAGCACAG-3′	5′-GGGTTCCATGGTGAAGTCAAC-3′
iNOS	5′-CAGGTGCTATTCCCAGCCCAACA-3′	5′-CATTCTGTGCAGTCCCAGTGAGGAA-3′
NF-κB	5′-GGCATGCGTTTCCGTTACAA-3′	5′-TGATCTTGATGGTGGGGTGC-3′
β-actin	5′-ACAGGATGCAGAAGGAGATTAC-3′	5′-AGAGTGAGGCCAGGATAGA-3′
